# Preparation and Performance Evaluation of a Self-Crosslinking Emulsion-Type Fracturing Fluid for Quasi-Dry CO_2_ Fracturing

**DOI:** 10.3390/gels9020156

**Published:** 2023-02-15

**Authors:** Jiani Hu, Meilong Fu, Minxuan Li, Yan Zheng, Guojun Li, Baofeng Hou

**Affiliations:** 1 Hubei Key Laboratory of Oil and Gas Drilling and Production Engineering, College of Petroleum Engineering, Yangtze University, Wuhan 430100, China; 2 CNOOC China Limited Hainan Branch, Haikou 570100, China; 3 Beijing AP Polymer Technology Co., Ltd., Beijing 102200, China

**Keywords:** Quasi-dry CO_2_ fracturing, self-crosslinking emulsion-type, comprehensive properties, enhanced oil and gas recovery

## Abstract

Quasi-dry CO_2_ fracturing technology is a new CO_2_ fracturing technology that combines liquid CO_2_ fracturing (dry CO_2_ fracturing) and water-based fracturing. It uses a liquid CO_2_ system containing a small amount of water-based fracturing fluid to carry sand, and it is characterized by sand blending at normal pressure, convenient preparation, the integrated application of resistance reduction and sand carrying, and no dedicated closed sand blender requirement. We developed a self-crosslinking emulsion-type water-based fracturing fluid (ZJL-1), which contained ionic bonds, hydrogen bonds, van der Waals forces, and hydrophobic associations, for quasi-dry CO_2_ fracturing, and the comprehensive properties of the ZJL-1 fracturing fluid were evaluated. The results showed that the ZJL-1 fracturing fluid had obvious viscoelastic characteristics, a heat loss rate of less than 10% at 200 °C, a good thermal stability, sufficient rheology under high temperature and high shear conditions, and a good thermal stability. The resistance reduction rate reached 70%, which demonstrates a good resistance reduction performance. Compared with conventional guar fracturing fluid, ZJL-1 can carry more sand and has a lower core damage rate. The on-site use of quasi-dry fracturing showed that optimizing the mixing ratio of liquid CO_2_ fracturing fluid and ZJL-1 fracturing fluid effectively enhanced oil and gas recovery. This can be used to optimize quasi-dry fracturing and can be used as a reference.

## 1. Introduction

In regard to oil and gas production, unconventional resources, such as shale gas and tight oil, have attracted much attention in the past decade and have become the focus in the development of energy resources by the global oil industry. According to the U.S. Energy Information Administration, global tight oil production will increase from 33% of the total land oil production to 51% in 2040 to alleviate the world’s growing energy demand and will account for nearly 10% of the global oil production. Low-porosity and low-permeability reservoirs, such as tight oil, gas, and shale gas reservoirs, are generally characterized by poor reservoir physical properties, a permeability generally lower than 0.1 mD, and natural productivity lower than industrial standards [1]. Appendix A Table A1 summarizes the complex properties of unconventional reservoirs, multi-stratum rock and fluid characterization, and rock mineralogy [2].

For unconventional oil and gas resources, the exploitation method necessarily has its particularities, and conventional hydraulic fracturing easily results in water sensitivity and water locking damages to reservoirs, which impacts the increases sought in oil production [3,4]. In recent years, dry CO_2_ fracturing has been developed at home and abroad. This technology uses 100% liquid CO_2_ as the fracturing fluid, thereby avoiding water sensitivity and water locking damage to reservoirs, and CO_2_ is discharged as gas during flowback without residue and damage [5,6,7].

For the Devonian water-sensitive shale formation in eastern Kentucky, the US Department of Energy (DOE) funded a liquid CO_2_ dry fracturing test. The test results showed that liquid CO_2_ dry fracturing was 1.9 times more effective than nitrogen fracturing and 4.9 times more effective than nitrogen foam fracturing [8]. Some researchers proposed increasing the flow rate of liquid CO_2_ to improve its sand carrying capacity, but increasing the flow rate of CO_2_ increases the friction loss in the pipeline [9,10]. Wang et al. conducted experiments to optimize different types of CO_2_ thickeners. The results showed that 1.5~2.0% concentration of TNJ-3 type thickeners increased the CO_2_ viscosity by 240~490 times and enhanced the performance of carbon dioxide fracturing fluids in TNJ-3 test evaluations [11]. The fatty acid thickener developed by Li Shan et al. can increase the viscosity liquid CO_2_ by 17–184 times, and the thickener has been successfully applied to construction sites in the Changqing gas field in the Ordos Basin for liquid CO_2_ fracturing, but because it is a nonpolar solvent with poor water solubility and low degradability, the thickener remains in the formation after compression and can cause reservoir damage [12]. The BJ company formed a CO_2_/N_2_ foam fracturing fluid system by mixing liquid N_2_ into liquid CO_2_ and used methoxy nonafluorobutane (C_4_F_9_OCH_3_) as a foaming agent, which retained the liquid CO_2_ fracturing fluid. However, the introduction of N_2_ greatly reduced the static pressure of the fracturing fluid system, which establishes higher requirements for the fracturing pump truck, thus limiting its application range [13,14,15]. Workers at the Changqing Oilfield, Jilin Oilfield, and Yanchang Oilfield have made attempts to use supercritical CO_2_ dry fracturing and have achieved certain results [16,17,18]. CO_2_ fracturing technology was applied in the Yanchang Oilfield 167 times for the production of tight oil, tight gas, and continental shale gas, and the production stimulation effect was remarkable [19]. However, conventional CO_2_ fracturing also has many problems, including low viscosity, poor sand-carrying and fluid-loss control capabilities, high frictional resistance, and difficulty in effectively creating fractures; moreover, special closed sand-mixing equipment is used, and the scale of sand addition is limited. As a result, the thickening, drag reduction, and sand-carrying properties of liquid CO_2_ are poor, and the ideal stimulation effect cannot be achieved; the fracturing equipment costs, material costs, and construction costs are too high for this method to be popularized and applied on a large scale [20,21].

Based on the above reasons, quasi-dry CO_2_ fracturing, which is a combination of liquid CO_2_ dry fracturing and conventional water-based fracturing, is currently being studied [22]. The quasi-dry CO_2_ fracturing technology proceeds as follows: CO_2_ thickener, water-based thickener, and proppant are added into water to form a water-based mixture with high viscosity and high sand ratio (water-based fracturing fluid accounts for 10–30%), the water-based mixture is blended with 90–70% liquid CO_2_ at the high pressure tee manifold of wellhead to form a mixed-phase liquid with some viscosity. Liquid CO_2_ is a weak solvent that is immiscible with clean water. The quasi-dry CO_2_ fracturing process requires the use of a liquid CO_2_ system containing a small amount of water-based fracturing fluid to carry sand. Moreover, the mixing ratio of liquid CO_2_ and water base is optimized. Under the condition of ensuring the stable performance of fracturing fluid, the dosage of the water-based fracturing fluid is required to be as low as possible and the damage to be minimal. This technology is characterized by low damage, no requirement for a dedicated closed sand blender, relatively simple operation flow and control, and suitability for large-scale sand addition and fracturing, etc., [23], and it exhibits the technical advantages of dry CO_2_ fracturing technology and meets the requirements for fracturing construction technology with the high sand ratio.

Traditional water-based fracturing fluid thickeners are mainly in the form of solid powders, and the solution dissolves slowly when used, which not only increases the workload but also increases the cost and causes the product to shear and degrade, affecting the use effect. It is also necessary to use a cross-linking agent for cross-linking to carry sand. The cross-linking effect is unstable and poorly controllable, resulting in a low sand ratio and insufficient sand-carrying capacity, resulting in unsatisfactory or even poor production enhancement effects. The integrated operation of low-concentration drag reduction and high-concentration sand-carrying cannot be well satisfied. Emulsion-type fracturing fluid thickeners, such as conventional polyacrylamide emulsions, are usually used in the preparation of low-concentration slick water. The amount of sand is less than that of low-sand, the cross-linking conditions are harsh, and it is difficult to prepare sand-carrying fluid. Therefore, this research develops a self-crosslinking, emulsion-based, water-based fracturing fluid system (ZJL-1) that satisfies the integration of drag reduction and sand-carrying and does not require a crosslinking agent for crosslinking. The fracturing fluid dissolves and self-crosslinks, and a strong spatial network structure is formed through ionic bonds, hydrogen bonds, van der Waals forces, and hydrophobic associations, so it has excellent shear resistance. It can still achieve a high sand ratio and sand carrying at high temperatures. A series of performance evaluations of the ZJL-1 fracturing fluid included studies of the temperature-resistance capacity, viscoelasticity, shear-resistance capacity, frictional properties, proppant suspension ability, gel breaking properties, and core damage properties. This fracturing fluid system can provide technical support for oilfield fracturing sites. Appendix A Table A2 compares the performance of the existing fracturing fluid systems on the market with the ZJL-1 fracturing fluid system.

## 2. Materials and Methods

### 2.1. Experimental Materials

**Figure 1:**
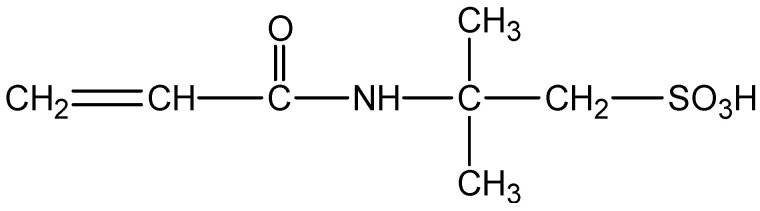
Molecular structure of AMPS.

**Figure 2:**
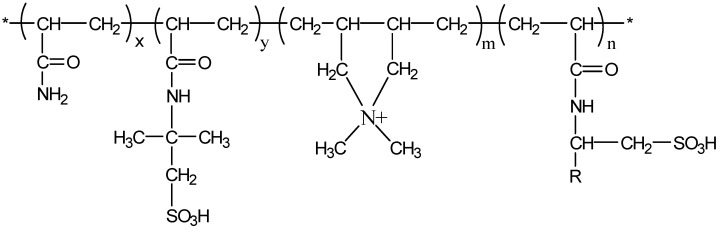
Molecular structure of the self-crosslinking emulsion thickener.

(1) Reagents: The self-crosslinking emulsion thickener was prepared by inverse emulsion polymerization. In a three-necked flask protected by nitrogen gas, add 140 g of silicone oil, 4 g of Span80 and 1 g of OP-10, and stir at high speed for 30 min. Dissolve 40 g of acrylamide, 10 g of 2-acrylamido-2-methylpropanesulfonic acid, and 5 g of dimethyl diallyl ammonium chloride in 140 g of deionized water. After the monomers are completely dissolved, slowly drop them into a three-necked flask, stir while adding dropwise, control the drop rate, finish the drop within 25–30 min, control the stirring speed, use an emulsifier to emulsify for 1.5 h, and keep the state of the water-in-oil emulsion stable. Add 0.05 g of benzoyl peroxide, 0.05 g of azobisisobutyronitrile, and 0.05 g of tert-butyl peroxide to the emulsion at 45 °C for initiation, raise the temperature of the reaction system to 85 °C, and control the reaction at this temperature 14 h after the reaction, the jacket of the reaction vessel was circulated through cooling water, and the temperature was lowered to 25 °C. The white emulsion obtained in the reaction vessel was a self-crosslinking emulsion type fracturing fluid thickener. After the reaction was completed and purified, the conversion rate was 95.6%. The structural formulas of AMPS and the self-crosslinking emulsion-based thickener are shown in Figure 1 and Figure 2. The degree of hydrolysis (mole fraction) and solid content were 23–27% and 88%respectively.

The intrinsic viscosity [η] of ZJL-1 was measured by a self-made dilution Ubbelohde viscometer, and the relative molecular mass was estimated according to the Mark-Houwink $\left[\eta \right]=KM^{\alpha }$ equation, where α = 0.8, K= 4.75 × 10^−3^ mg/L, the relative molecular mass M is 8.14 × 10^6^ [24]. The self-crosslinking emulsion thickener was mixed with mass fractions of 0.5 wt%, 1.0 wt%, and 1.5 wt% in water and mechanically stirred at 600 r/min at room temperature until completely dissolved to form three different concentrations. The ZJL-1 fracturing fluid system, without special instructions, and the subsequent ZJL-1 fracturing fluids all used simulated formation water.

Acrylamide, 2-acrylamido-2-methylpropane sulfonic acid (AMPS), dimethyl diallyl ammonium chloride (DMDAAC), and silicone oil were all purchased from Macklin’s Reagent Co., Ltd., Shanghai, China. Span80, OP-10, benzoyl peroxide, azobisisobutyronitrile, and tert-butyl peroxide were purchased from Aladdin Biochemical Technology Co., Ltd., Shanghai, China.

Nitrogen (purity 99.95%) was provided by Xinxing Gas Co., Ltd., Wuhan, China. China. All of the above reagents were of industrial purity.

(2) The proppant sample was low-density ceramsite, with particle sizes of 0.2–0.4 mm and densities of 1.0–1.3 g/cm^3^.

(3) Conventional guar fracturing fluid was provided by Yanchang Oilfield.

(4) Synthesized brine was used in the present study, and the ionic composition is shown in Table 1.

(5) Core for experiments: a long core spliced with natural cores was provided by S Oilfield through artificial single seam splicing; the basic physical properties of the natural cores are shown in Table 2.

### 2.2. Experimental Methods

#### 2.2.1. IR Analyses

Using a Bruker Tensor 27 (Salbrücken, Germany) Fourier Transform Infrared Spectrometer, the liquid to be measured was characterized in the infrared by applying drops of the liquid to a pure potassium bromide pressed tablet [25].

#### 2.2.2. SEM Analyses

The surface morphology of ZJL-1 was investigated after self-crosslinking using a Thermo Nicolet 380 Scanning Electron Microscope (Massachusetts, USA). ZJL-1 was frozen at −20 °C and then freeze-dried for 24 h. Before SEM observation, the freeze-dried samples were cut open to expose the internal structure of the cross-section, sprayed with gold and then observed [26].

#### 2.2.3. Thermogravimetric Analyses (TGA)

The temperature was raised from 25 °C to 500 °C with a Perkin-Elmer Pyris Diamond TG thermal analyzer (Massachusetts, USA) to analyze the thermal stability of ZJL-1. First, glass slides were wiped clean, and then appropriate amounts of sample was applied to them. Then, the glass slides were baked in a drying oven at 60 °C for a period of time to remove the moisture in the samples. Nitrogen was used as the protective gas, and the heating rate was 10 °C/min.

#### 2.2.4. Test of Viscoelasticity

Tests of viscoelasticity and rheological properties were carried out with a Haake RS6000 rheometer (Karlsruhe, Germany). The storage modulus (G′) and loss modulus (G″) of ZJL-1 were determined at different concentrations, with the temperature set to 25 °C, the 35 mm cone-and-plate test system and oscillating measurement mode selected, the oscillating angular frequency set to 1 Hz, and the yield stress set to 0.5 Pa [27].

#### 2.2.5. Tests of Temperature and Shear Resistance

The experiment used a HaakeMars60 rheometer (Karlsruhe, Germany). The reactor inside the rheometer was a steel cylinder, and the pressure of the reactor was 40 MPa, which met the requirements of this experiment. ZJL-1 was poured into the reaction kettle, the temperature control line and the pressure sensing line were connected, the kettle was heated to 160 °C with the external circulation of the water bath, nitrogen was injected into the reaction kettle, the pressure was adjusted to 20 MPa, and the shear speed was set to 170 s^−1^ for 2 h. The relationship between the apparent viscosity of the fracturing fluid and the time curve was measured [28,29]. Fracturing fluid must have a viscosity greater than 20 mPa·s to meet the requirements of fracturing fluid transportation proppants [30,31,32].

#### 2.2.6. Test of Gel Breaking Property

The ZJL-1 fracturing fluid was sealed in a container and placed in a constant temperature water bath at 90 °C, and kerosene was added to stir and break the gel. A ZNN-D6B six-speed rotational viscometer (Qingdao, China) was used to measure the viscosity of the fracturing fluid before and after breaking the gel and record the break time. A DT-102A automatic interfacial tension meter (Zibo, China) was used to measure the surface interfacial tension of the breaking fluid, and the breaking performance of the ZJL-1 fracturing fluid was investigated [33].

#### 2.2.7. Method for the Frictional Drag Tests

**Figure 3:**
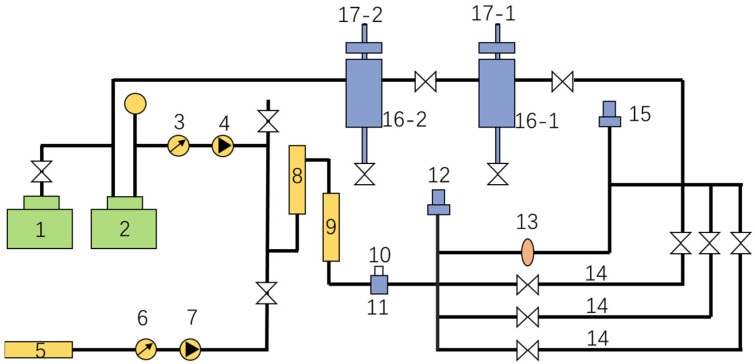
Test device for measuring frictional drag in high-temperature and high-pressure fluid circulation loops. 1—Fluid pressure pump; 2—high pressure container; 3—flow meter; 4—injection pump; 5—thickener storage tank; 6—flow meter; 7—injection pump; 8—blending tank; 9—pipe heater; 10—mass flow meter; 11—pressurizing pump; 12—inlet pressure sensor; 13—pipe viewport; 14—test pipes with diameters of 6 mm, l0 mm, and 14 mm, respectively; 15—outlet pressure sensor; 16—separator; 17—temperature and pressure regulators.

Frictional drag property tests were carried out with a large-sized high-temperature and high-pressure fluid circulation device, as shown in Figure 3. The frictional drag in pipe flow was tested before and after the thickener was added into the ZJL-1 fracturing fluid, the differential pressure in the pipeline was calculated using the differential pressure method for pipe flow in a high-pressure long pipe, and the drag reduction efficiency was calculated with Formula (1).

Dr = (∆P_1_ − ∆P_2_)/∆P_1_ × 100% (1)

where Dr is the drag reduction efficiency, ∆P_1_ is the differential pressure when water flows through the pipeline, and ∆P_2_ is the differential pressure when the ZJL-1 fracturing fluid with added thickener flows through the long pipe. The method employed a pipe with an internal diameter of 10 mm and a test pipeline with a length of 2 m, and the drag reduction efficiency values were tested with different concentrations of thickener added at different flow rates at a temperature of 25 °C and a pressure of 15 Mpa [34,35].

#### 2.2.8. Method for Testing Sand Suspension Properties

A static sand suspension visible reactor was used. ZJL-1 was fully dissolved in water with stirring. Settling of the proppant in the ZJL-1 fracturing fluid with different thickener concentrations was observed using the indoor static observation method [36], and the settling speed of the proppant in the ZJL-1 fracturing fluid was analyzed. The experimental temperature was 25 °C, the pressure was 15 MPa, 30/50 mesh ceramsite proppant was selected, the density of the proppant was 1.3 g/cm^3^, and the rotational speed of the stirrer was 1000 rpm [37].

#### 2.2.9. Method for Testing Core Damage

**Figure 4:**
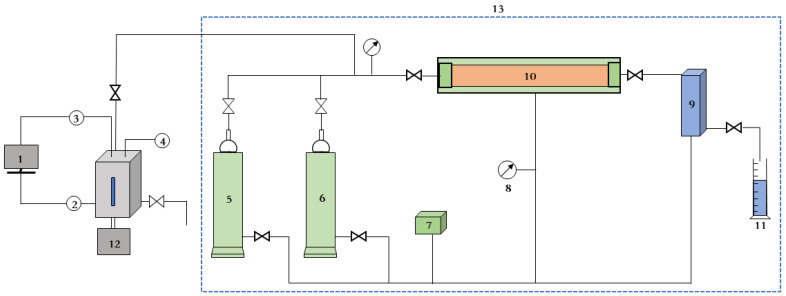
Core damage test system. 1—Temperature and pressure control system; 2—temperature sensor; 3—pressure transducer; 4—magnetic sensor; 5—oil; 6—formation water; 7—constant-flux pump; 8—confining pressure; 9—backpressure valve; 10—core gripper; 11—volumetric cylinder; 12—electrical machine; 13—container.

Evaluations of core damage were carried out with a fracturing fluid-caused damage test device [38], as shown in Figure 4. The core permeability was determined before and after the occurrence of damage caused by the fracturing fluid, and the core damage ratio could be obtained by substituting the determined permeability values into the matrix permeability damage ratio Formula (2).

(2) $$\eta_{d}=K_{1}-K_{2}/K_{1}\times 100\%\;$$

where $\eta_{d}$ is the matrix permeability damage ratio and K_1_ and K_2_ are the permeability values before and after core damage, respectively, used to evaluate the extent of damage to the formation caused by the fracturing fluid [39].

## 3. Results and Discussion

### 3.1. Infrared Spectroscopic Analyses

**Figure 5:**
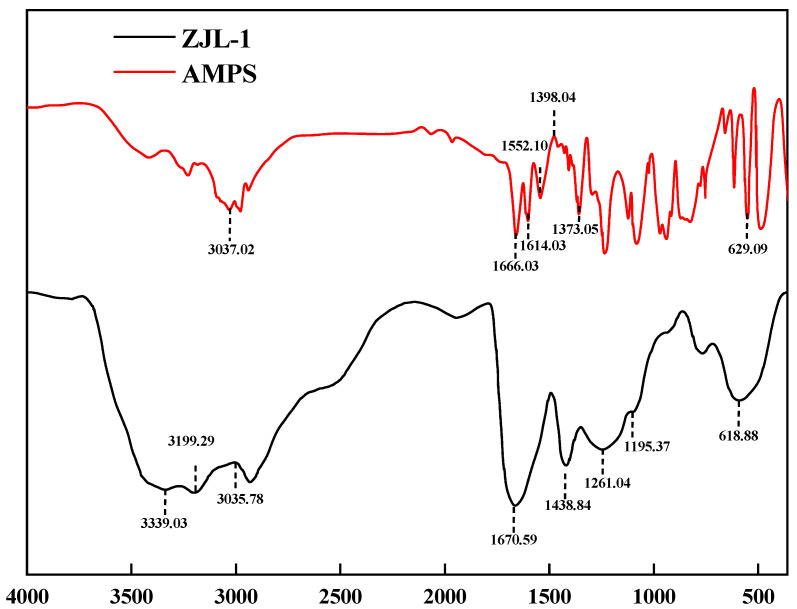
FTIR spectra of AMPS and ZJL-1.

Figure 5 shows the infrared spectrum of AMPS, which exhibited a vinyl =C-H stretching vibration peak at 3037.02 cm^−1^, a C=C stretching vibration peak at 1666.03 cm^−1^, and secondary vibrational peaks at 1614.03 cm^−1^ and 1552.10 cm^−1^. The characteristic amide II band and amide I absorption bands appeared at 1373.05 cm^−1^ and 1398 cm^−1^ for the branched -CH, C-H in-plane bending vibrations, and the peak at 629.09 cm^−1^ was a characteristic absorption peak for a sulfonic acid group. In the infrared spectrum of ZJL-1, there were two primary amide peaks at 3199.29 cm^−1^ and 3339.03 cm^−1^, a peak at 3035.78 cm^−1^ for the stretching vibration of the vinyl =C-H, a peak at 1670.59 cm^−1^ for C=C stretching vibration, a sulfonic acid symmetrical stretching vibration peak at 1438.84 cm^−1^, a secondary amide II stretching vibrational peak at 1261.04 cm^−1^, a tertiary ammonium vibrational peak at 1195.37 cm^−1^, and the peak at 618.88 cm^−1^ 1 is characteristic of the sulfonic acid group; the absorption peaks of the AMPS monomer functional groups appeared in the infrared spectrum of ZJL-1, which confirmed that ZJL-1 was chemically modified by AMPS.

### 3.2. Scanning Electron Microscope Tests

**Figure 6:**
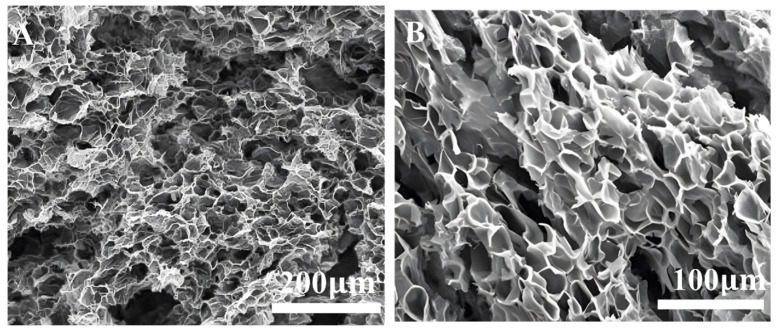

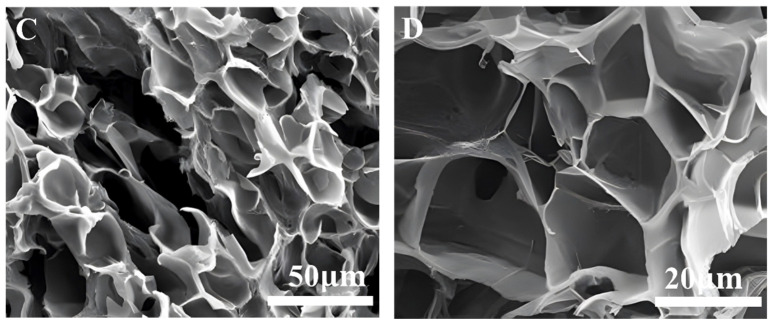
SEM images of ZJL-1. (**A**). 200 Um; (**B**). 100 um; (**C**). 50 um; (**D**). 20 um.

Figure 6 shows a scanning electron microscopy image of the 0.5% ZJL-1 base fluid, and the aggregation state of the molecular main chain is clearly observed. The molecular chain was stretched in the solution to form a more compact and complex spatial network. Many small branch chains were intertwined with each other to form an associated structure, which increased the hydrodynamic volume and the viscosity of the solution.

**Figure 7:**
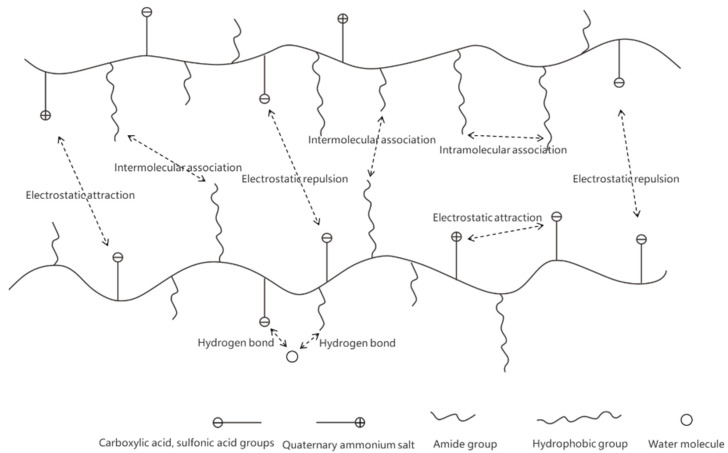
Associations in the ZJL-1.

After ZJL-1 was dissolved in water, the carboxyl, sulfonic, and quaternary ammonium salt groups in and between the molecular chains of the copolymer underwent self-crosslinking through ionic bonds, the quaternary ammonium groups in the molecular chains and the sulfonic groups of the anionic emulsifier dissolved in water to form secondary self-crosslinks through ionic bonding, and the long-chain hydrophobic groups in the molecular chains and the nonionic groups of the nonionic emulsifier underwent tertiary self-crosslinking through van der Waals forces and hydrophobic association. In addition, there were strongly polar hydrogen bonds, including H-O bonds and H-N bonds, in the molecular chains, which enhanced the self-crosslinking [40,41,42]. Therefore, ZJL-1 can form a three-dimensional network structure through weak associations, such as hydrogen bonds, ionic bonds, hydrophobic associations, and van der Waals forces, showing good viscoelasticity, as shown in Figure 7. No added crosslinker was needed after dissolution, and various properties were evidently better than those obtained with crosslinked fracturing fluid. This self-crosslinking mode was completely different from other crosslinking modes involving added crosslinker due to the strong covalent bonds (polar covalent bonds, coordinate covalent bonds, etc.,). Crosslinking via chelating bonds is a unique and innovative crosslinking mode [43].

### 3.3. TGA Test Results

**Figure 8:**
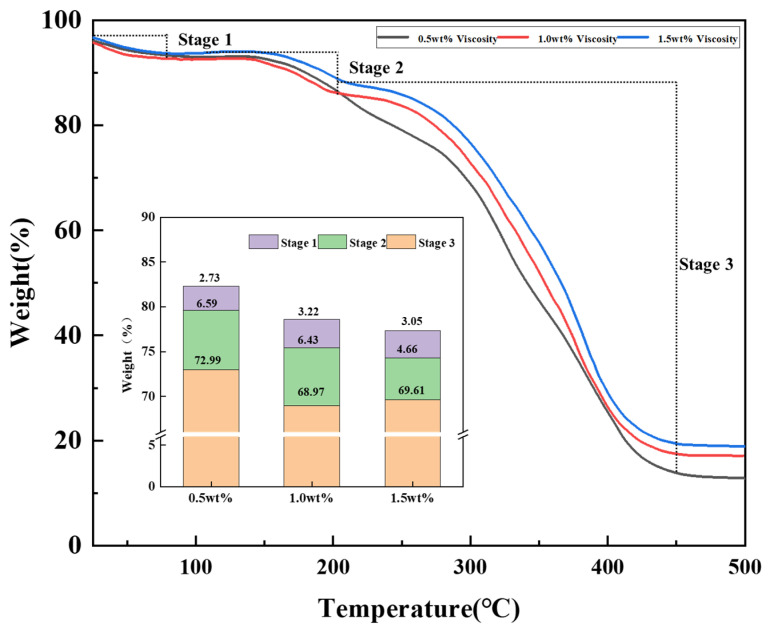
Thermogravimetric curves for different concentrations of ZJL-1.

As shown in Figure 8, the thermal decomposition temperatures of ZJL-1 were analyzed by using concentrations of 0.5 wt%, 1.0 wt%, and 1.5 wt%, and the graphs show that ZJL-1 went through three weight loss stages for each concentration profile. The first stage occurred between 25 and 80 °C, and the weight loss was generally low; ZJL-1 was ground into a powder and absorbed moisture from the air prior to testing, so this weight loss was due to the volatilization of moisture. When the temperature rose to 80–200 °C, the second weight loss occurred and was mainly due to thermal decomposition of the amide and sulfonic acid groups in the polymer, and the third stage of weight loss occurred between 200 and 450 °C due to thermal decomposition of -SO_3_H in ZJL-1, which is the largest weight loss observed. As the concentration of ZJL-1 was increased, the total amount of mass lost decreased, with the smallest total mass loss for 1.5 wt% ZJL-1, 7.71%, occurring between 25 and 200 °C. A thermal weight loss of less than 10% for the polymer indicates that the substance is able to withstand the corresponding temperature. The apparent viscosity of ZJL-1 is closely related to the density of the spatial mesh structure, the bond length between molecules, and the entanglement strength of the spatial mesh structure. With the increase of temperature, the intermolecular chemical bonds are gradually broken and this formed reticular structure is gradually destroyed due to the increase of molecular activity. Therefore, the results of the thermogravimetric analyses indicated that ZJL-1 can withstand temperatures up to 200 °C. On the one hand, the existence of the network structure delays the thermal degradation of ZJL-1, and on the other hand, the sulfonic acid group increases the temperature resistance of ZJL-1. The better temperature resistance of ZJL-1 is conducive to maintaining better rheological properties in high temperature environments [44].

### 3.4. Viscoelasticity Test Results

**Figure 9:**
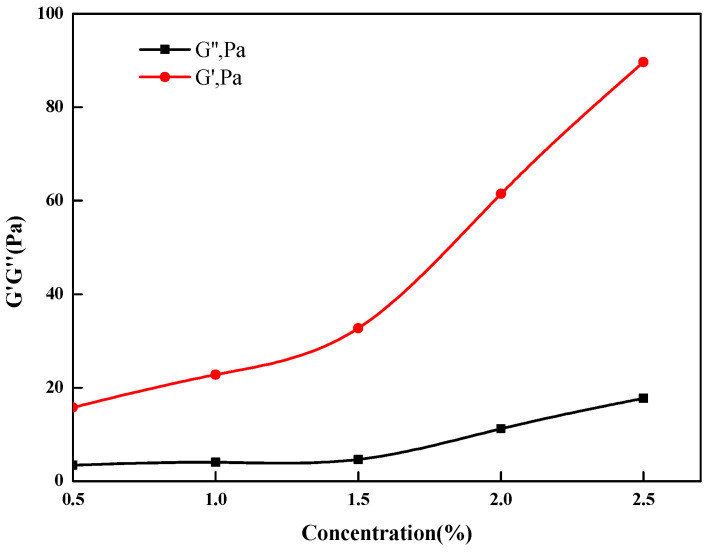
Viscoelasticities at different thickener concentrations.

G′ reflects the elastic behavior of a polymer caused by its reticular structure. Decreases in G′ indicate that the elastic behavior of the polymer is weakening. When G′ > G″, it indicates that the polymer exhibits relatively strong elastic behavior. The hydrophobic groups introduced into the molecular structure produce hydrophobic associations, which causes the molecular chains to intertwine, the spatial structure becomes more complex, and the elasticity of the solution is enhanced [45]. With increases in the thickener concentration, intermolecular association of hydrophobic monomers is enhanced so the hydrodynamic volume of the fracturing fluid increases and the dynamic modulus increases. As shown in Figure 9, each G′ value was higher than the corresponding G″ value in the test concentration range, indicating evident viscoelasticity and stronger spatial reticular structures. The elastic resistance prevents settling of the proppant and improves the sand carrying capability of the fracturing fluid [46].

### 3.5. Temperature and Shear Resistance Tests

**Figure 10:**
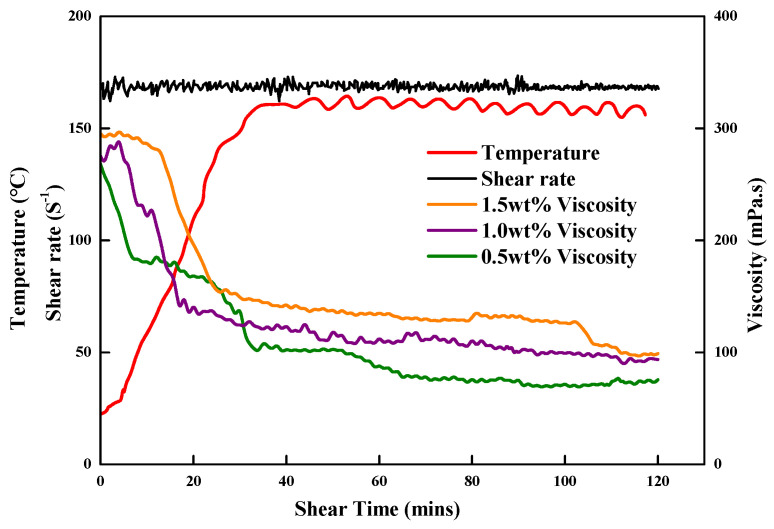
Shear resistance tests of the ZJL-1 fracturing fluid.

As shown in Figure 10, at 160 °C and 20 MPa, when the concentration of the added ZJL-1 was 0.5%, as the shear time increased, the viscosity of the fracturing fluid remained at 167.84 mPa·s for 20 min before shearing and then gradually decreased and finally stabilized at 73.94 mPa s. When the ZJL-1 concentration was 1.0%, the viscosity decreased with increasing shear time and finally stabilized at 93.65 mPa·s. When the concentration of ZJL-1 was 1.5%, the fracturing fluid viscosity reached a maximum value of 290 mPa·s, remained at 195.84 mPa·s 20 min before shearing, and finally showed a downward trend but remained at approximately 100 mPa·s overall. At low concentrations, the molecular chains of ZJL-1 tend to associate intramolecularly to form separate molecular micelles. The increase in hydrodynamic radius is small, so the increase in viscosity is low. As the concentration increases, intermolecular association begins. The hydrodynamic radius of the polymer increases rapidly, and when the concentration exceeds a certain level, the intermolecular association of ZJL-1 dominates, which promotes the formation of the polymer network structure, and the hydrodynamic radius increases significantly, resulting in a rapid increase in viscosity; at the same time, because this weak association is a dynamic equilibrium state of continuous formation and destruction, at high shear rates, the weak association structure is largely destroyed, and the viscosity of the polymer decreases; after the shear rate decreases, the destroyed weak association The cooperation gradually recovered and the viscosity of the polymer increased [47]. When the fracturing fluid was sheared at different concentrations, the viscosity was maintained above 70 mPa·s, so the fracturing fluid exhibited good temperature resistance and shear resistance and meets the sand-carrying capacity requirement at this temperature.

### 3.6. Gel Breaking Properties

The gel breaking time was 30 min, and the gel breaker exhibited a milky white color without suspended solid matter. The supernatant of the gel breaker was taken for testing. The viscosity of the gel breaker was determined at 90 °C as 1.7 mPa·s, the surface tension of the gel breaker was 22.7 mN/m, and the interfacial tension between the gel breaker and dehydrated kerosene was 1.06 mN/m. After gel breaking, the fracturing fluid exhibited low viscosity, surface tension, and interfacial tension, which favored rapid and thorough flowback after construction [48].

### 3.7. Frictional Drag Properties

**Figure 11:**
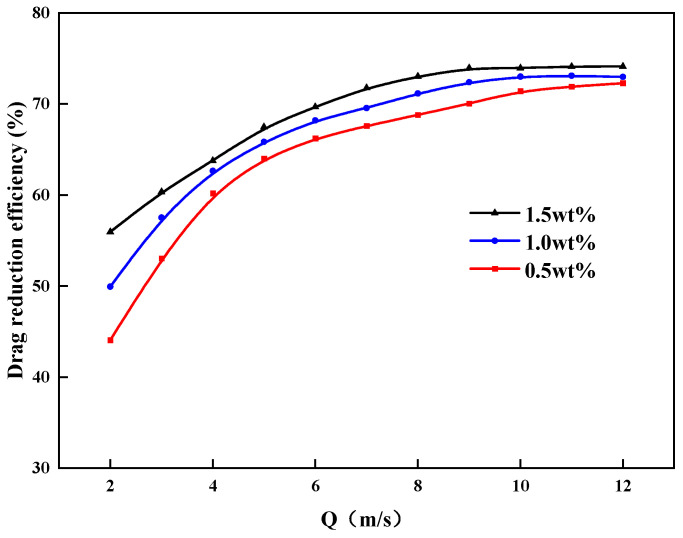
Plots of drag reduction efficiency versus displacement.

As shown in Figure 11 for indoor study conditions, the drag reduction efficiency of the ZJL-1 fracturing fluid system increased gradually with increasing flow rate and added thickener concentration. When the thickener concentration was 0.5% and the flow rate was 12.0 m^3^/min, the drag reduction efficiency of the fracturing fluid system reached 72%. The trends for changes in drag reduction efficiency shown in the figure indicate that the ZJL-1 fracturing fluid system meets the requirement for low frictional drag in field construction and is an excellent drag-reduction and thickening fracturing fluid system. Research has shown that a simple polymer long-chain structure is easy to break under high shear and cannot achieve a long-lasting lowering effect, so thickening agent has side groups introduced. The molecular chain forms a spiral structure that can be fully extended in water and has a certain elasticity, and its solutions form elastic bottom layers in the pipeline to lower the resistance.

### 3.8. Sand Suspension Properties

**Figure 12:**
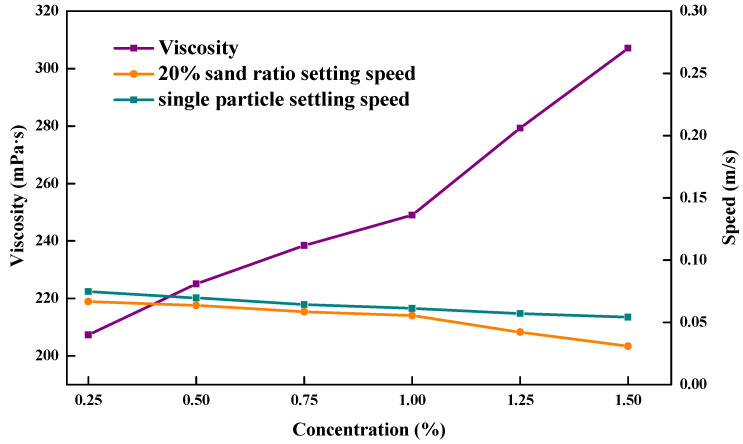
Relationships between the ZJL-1 fracturing fluid concentration and sedimentation rate and viscosity.

**Figure 13:**
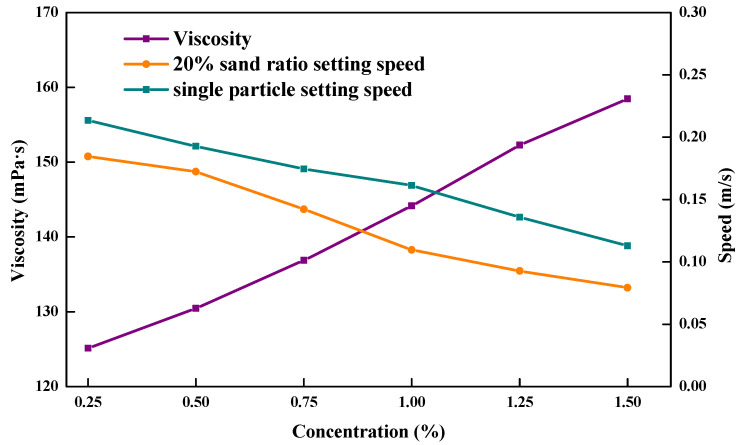
Relationships between the concentration of conventional guar fracturing fluid and the sedimentation rate and viscosity.

The falling times and settling speeds were determined at 90 °C and 15 MPa with a proppant settling test device for the single-particle proppant, the proppant with a sand ratio of 20% in the ZJL-1 fracturing fluid, and conventional guar fracturing fluid. Figure 12 and Figure 13 show that in the test done at 90 °C, increases in the mass fraction of thickener caused the sand settling speed for the single-particle ceramsite in the ZJL-1 fracturing fluid to decrease gradually from 0.082 mm/s to 0.056 mm/s, which was only approximately half the rate seen for conventional guar fracturing fluid. However, the single-particle proppant only indirectly reflects the sand-carrying performance of the fracturing fluid. The sand-carrying time test results for the fracturing fluid containing a 20% sand ratio showed that the settling speed gradually decreased from 0.071 mm/s to 0.031 mm/s with increases in the mass fraction of thickener, which was significantly lower than that of guanidine gel fracturing fluid; therefore, the self-crosslinked emulsion-type fracturing fluid exhibited good sand-carrying performance. During practical application, the sand-carrying fluid flows in the wellbore and fracture, and the settling speed of the proppant is lower than that suggested by static sand-carrying experimental data because it is subjected to a strong shearing effect.

### 3.9. Evaluation of Core Damage

Calculation performed with the experimental results yielded permeabilities and core damage ratios, as shown in Table 3. According to the industry standard SY/T 5358-2010 “Formation damage evaluation by flow test”, the extent of core damage degree caused by the ZJL-1 fracturing fluid was low, with a mean damage ratio of 14.055%, and the mean damage ratio of conventional guar fracturing fluid is 33.075%, indicating that this fracturing fluid can reduce the damage done to reservoirs and meet the field construction requirement.

## 4. Field Application

### 4.1. Basic Situations of the Experimental Well

The fracturing target layer in Well XX-Xie 1 has a burial depth of 3446 m, a porosity range of 7.19–21.84%, and a permeability range of 0.03–50 mD with an average of 2.4 mD. It is dominated by fine sandstone, has a clay mineral content of 14.2%, is characterized by low porosity and low permeability overall, and is a reservoir with moderate to strong water sensitivity. The flowback rate after conventional fracturing is 50–60%, so the postfracturing effect is not satisfactory. To reduce the extent of reservoir damage, quasi-dry CO_2_ fracturing technology was used with this well. ZJL-1, hydrophobic long-chain ester-based thickener, proppant, gel breaker, etc., were added to clean water to form the water-based system with high viscosity and high sand ratio (the proportion of water-based fracturing fluid was 30%), which was blended with liquid CO_2_ (proportion of 70%). The designed total construction displacement was 6.1–7.3 m^3^/min, the liquid CO_2_ injection displacement was 4.3–5.2 m^3^/min, the ZJL-4 fracturing fluid system injection displacement was 1.8–2.1 m^3^/min, and the selected proppant was 40–70 mesh ceramsite.

### 4.2. Construction Process and Effect Analysis

**Figure 14:**
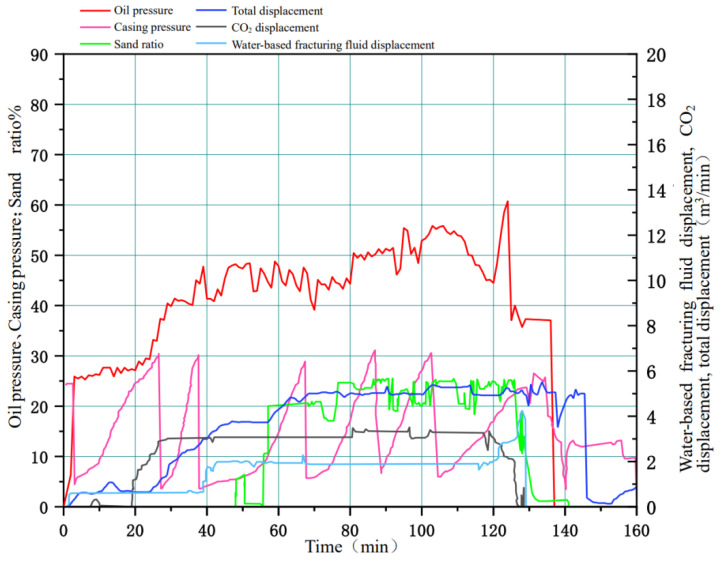
Fracturing construction parameters and construction curves.

In July 2020, quasi-dry CO_2_ fracturing construction was carried out smoothly at S Oilfield Well XX-Xie 1. The process flow was divided into two parts: one part involved pressurizing liquid CO_2_ and pumping it to the wellhead, and the other part involved blending clean water, ZJL-1, a hydrophobic long-chain ester-based thickener, proppant and gel breaker with a fracturing blender truck to form the proppant mortar with high viscoelasticity, which was pumped to the wellhead. These two pumped liquids converged at the tee of the wellhead, formed a mixed liquid phase with sufficient viscosity and structure under turbulent conditions to carry out fracturing construction with integrated drag reduction and sand carrying. In this construction, the amount of liquid CO_2_ fracturing fluid consumed was 350 m^3^ in total, the amount of ZJL-1 fracturing fluid consumed was 150 m^3^, the amount of sand added was 40 m^3^, the construction pressure was 25–60 MPa, the fracturing pressure was 47.5 Mpa, the liquid CO_2_ fracturing fluid injection displacement was 4.5 m^3^/min, the ZJL-1 fracturing fluid injection displacement was 2.0 m^3^/min, the shut-in pressure was 29 Mpa, the mean sand ratio was 18%, and the maximum sand ratio was 25%. The actual construction parameters and the design parameters were relatively highly matched, and the fracturing construction parameters and construction curves are shown in Figure 14. After the fracturing measurements were taken, the daily fluid production was determined to be 16.5 m^3^, the daily oil production was 10.2 m^3^, the daily gas production was 2.5 × 10^4^ m^3^, the flowback period was 10 days, and the flowback rate was 120%. The field application showed that the production rate of this well was increased with this fracturing mode as compared with the use of the previous fracturing mode. The daily oil production per single well doubled, and the daily gas production increased by a factor of 6.5 with the evident oil production increase.

## 5. Conclusions

In this study, we developed a self-crosslinking emulsion-type ZJL-1 fracturing fluid for quasi-dry CO_2_ fracturing, which formed a strong spatial network structure involving ionic bonds, hydrogen bonds, van der Waals forces, and hydrophobic associations, so self-crosslinking was enhanced without the need for additional crosslinkers.

The heat loss rate of the ZJL-1 fracturing fluid was less than 10% at 200 °C, and it showed good thermal stability and obvious viscoelasticity. After shearing for 2 hours at 160 °C with a shear rate of 170 s^−1^, the viscosity of the ZJL-1 fracturing fluid was still higher than 70 mPa s, indicating good shear resistance. The drag reduction rate reached 70%, indicating good drag reduction performance. Compared with the conventional guar fracturing fluid, the ZJL-1 fracturing fluid showed better sand-carrying performance and caused less damage to the core permeability. Field tests showed that upon optimizing the mixing ratio for the liquid CO_2_ fracturing fluid and ZJL-1 fracturing fluid, the increases in gas and oil production were obvious. This was beneficial for environmental protection and reservoir protection and met the requirements of integrated application for resistance reduction and sand carrying, and it has broad application prospects.

## Figures and Tables

**Table 1:** Ionic element composition of the synthesized brine used.

Ion	Concentration, mg/L
Na^+^	7012.05
Ca^2+^	500.86
Mg^2+^	341.26
Cl−	11,398.45
total	19,252.62

**Table 2:** Related physical property parameters of natural short cores.

No.	Length (cm)	Diameter (cm)	Permeability (mD)	Porosity (%)	Mean Permeability (mD)	Mean Porosity (%)
1^#^	6.086	2.327	0.2156	7.24	0.386	8.65
2^#^	6.968	2.331	0.2221	7.87		
3^#^	6.552	2.328	0.4507	8.33		
4^#^	6.724	2.326	0.4330	9.26		
5^#^	6.414	2.338	0.6115	10.55		

**Table 3:** Core damage properties of the ZJL-1 fracturing fluid.

Fracturing Fluid System	Temperature (℃)	Pressure (MPa)	Injection Quantity (%)	Permeability K_1_ (mD)	Permeability K_2_ (mD)	Core Damage Ratio D_d_ (%)	Mean D_d_ (%)
1.5 wt% ZJL-1 fracturing fluid	20	8	0.5	0.382	0.331	13.350	14.055
	20	8	1.0	0.388	0.329	15.206	
	20	8	1.5	0.386	0.335	13.212	
	40	15	0.5	0.389	0.346	11.053	
	40	15	1.0	0.383	0.323	15.665	
	40	15	1.5	0.385	0.324	15.844	
1.5 wt% Conventional guar fracturing fluid	20	8	0.5	0.385	0.509	32.43	33.075
	20	8	1.0	0.385	0.510	32.56	
	20	8	1.5	0.382	0.508	33.17	
	40	15	0.5	0.381	0.508	33.54	
	40	15	1.0	0.388	0.519	33.80	
	40	15	1.5	0.384	0.510	32.95	

## Data Availability

The data presented in this study are available in the insert article.

## Appendix A

**Table A1:** Typical rock/formation and fluid properties of shale oil reservoirs.

Reservoir Formation, Rock and Fluid Properties	Typical Range (Collected from Literature)
Permeability	1E-5-0.1 mD
Porosity	2–18%
Reservoir temperature	200–240°F
Formation pressure	3000–8000 psi
Saturation pressure	2500–3500 psi
Ney pay thickness	8–2600 ft
Formation depth	2000–14,000 ft
Drive mechanism	Poor sweep and low-pressure connectivity
Initial water saturation	25–50%
Pressure gradient	0.42–0.7 psi/ft
Rock type	Mixed-silt, limestone, sand & shale
Thermal maturity (R_o_)	0.6–1.8%
Wettability	Mixed to oil-wet
Contact angle	80°–145°vvv
Oil–water interfacial tension (IFT)	17–34 mN/m
Natural fracture intensity	0–32 per ft
Clay content	7–30%
Total organic content	0.1–12%
Bulk density	2.3–2.5 g/cm^3^
Grain density	2.5–2.7 g/cm^3^
Rock grain size	Below 62.5 μm
Average pore radius	0.01–0.03 μm
Oil density	38–42 API
Oil viscosity	Below 4.2 cP
Gas oil ratio (GOR)	500–1800 scf/stb
Oil polarity	More towards paraffinic
Fluid pH	Acidic
Total acid number	0.02–0.36 mg KOH/g
Total base number	0.12–1.16 mg KOH/g
Brine specific gravity	Heavy
Brine salinity	High
Brine total dissolved solids (TDS)	228,500–285,000

**Table A2:** Performance comparison table of fracturing fluid systems on the market and the ZJL-1 fracturing fluid system.

Fracturing Fluid System	Producer	Drag Reduction Rate/%	Viscosity of Gel Breaking Liquid/mPa.s	Temperature Resistance, Shear Resistance	Reservoir Damage Rate/%
FR1-1 fracturing fluid system	Shandong Ruixing Petroleum Technology Service Co., Ltd.	40	3.7~4.8	150 °C, 170 s^−1^ for 2 h, 40 mPa.s	30
JNBY fracturing fluid system	Jinan Beyate Chemical Co., Ltd.	40	3.5	120 °C, 170 s^−1^ for 2 h, 50 mPa.s	26
Guar gum fracturing fluid system	Henan Yuanchun Chemical Co., Ltd.	60	<5	45 °C, 170 s^−1^ for 1.5 h, 100 mPa.s	18
Guar gum fracturing fluid system	Hubei Dongsao Chemical Technology Co., Ltd.	60	<5	90 °C, 170 s^−1^ for 2 h, 70 mPa.s	25
Environmental protection emulsion fracturing fluid system	Henan Tianxiang New Material Co., Ltd.	55	4	70 °C, 170 s^−1^ for 2 h, 80 mPa.s	15
BS-1A fracturing fluid system	Zhejiang Fenghong New Material Co., Ltd.	65	<5	40 °C, 170 s^−1^ for 2 h, 100 mPa.s	25
ZL-1 fracturing fluid system	Lab homemade	72	1.7	160 °C, 170 s^−1^ for 2 h, 100 mPa.s	14
